# Functional architecture of the foveola revealed in the living primate

**DOI:** 10.1371/journal.pone.0207102

**Published:** 2018-11-28

**Authors:** Juliette E. McGregor, Lu Yin, Qiang Yang, Tyler Godat, Khang T. Huynh, Jie Zhang, David R. Williams, William H. Merigan

**Affiliations:** 1 Center for Visual Science, University of Rochester, Rochester, New York, United States of America; 2 Institute of Optics, University of Rochester, Rochester, New York, United States of America; 3 Department of Biomedical Engineering, University of Rochester, Rochester, New York, United States of America; 4 Flaum Eye Institute, University of Rochester Medical Center, Rochester, New York, United States of America; National Eye Centre, UNITED STATES

## Abstract

The primate foveola, with its high cone density and magnified cortical representation, is exquisitely specialized for high-resolution spatial vision. However, uncovering the wiring of retinal circuitry responsible for this performance has been challenging due to the difficulty in recording receptive fields of foveal retinal ganglion cells (RGCs) *in vivo*. In this study, we use adaptive optics scanning laser ophthalmoscopy (AOSLO) to image the calcium responses of RGCs in the living primate, with a stable, high precision visual stimulus that allowed us to localize the receptive fields of hundreds of foveal ganglion cells. This approach revealed a precisely radial organization of foveal RGCs, despite the many distortions possible during the extended developmental migration of foveal cells. By back projecting the line connecting RGC somas to their receptive fields, we have been able to define the ‘physiological center’ of the foveola, locating the vertical meridian separating left and right hemifields *in vivo*.

## Introduction

Visual abilities such as reading and face recognition are reliant on the specialized structures of the human fovea. Foveal specializations include a very high density of cone photoreceptors with displacement of inner retina neurons, as well as their vascular support, hundreds of microns away from the foveal center. The most central cones are located in the foveola, a 350 μm rod-free region comprising the floor of the foveal depression [1]. An equally important specialization is the high degree of thalamic and cortical magnification that assigns a disproportionate fraction of central visual pathways to central retina, facilitating the fine spatial scale discrimination for which foveal vision is known [2]. Functional studies of central vision therefore require both an animal model that shares these features and an experimental paradigm that can present high spatial frequency, high precision stimuli.

Human-like foveal specialization is present in the retina of great apes and old world primates such as the macaque, and less faithfully in new world primates such as marmosets and squirrel monkeys, making these animal models critical to understanding foveal function. Other animal models routinely used in vision research including cat, dog, rodent, rabbit etc., do not share the fine spatial scale or other specializations of human fovea [3,4].

The microanatomy of the primate fovea has been characterized by both light and electron microscopy studies of fixed and sectioned tissue [5–9] however these focused primarily on more eccentric foveal locations (200 to 2400 μm in macaque) compared to the present study (0 to 200 μm). Furthermore, histological data are necessarily structural rather than functional and are subject to a range of preparation artifacts including tissue shrinkage, distortion during embedding and the challenges of reconstructing sectioned tissue.

Foveal retinal ganglion cells have been studied functionally *in vivo* by extracellular single unit recording from the lateral geniculate nucleus (LGN). This technique requires glass insulated tungsten microelectrodes [10,11] or saline filled micropipettes [12] to be inserted into the LGN through a craniotomy. Recordings have been made directly from retinal ganglion cells *in vivo* by inserting an electrode through a cannula entering the eye behind the limbus [13–15]. Both approaches require substantial surgery and mapping the responses of large numbers of cells is a slow and painstaking process. More recently multi-electrode arrays [16] have been adopted to increase the efficiency of data collection, however this requires an *in vitro* preparation with the retina isolated from the choroid and pigment epithelium and perfused in a recording chamber under a microscope. Preparations of this type have also allowed patch clamp recording from foveal retinal ganglion cells [17] but this approach has proven difficult to implement at the foveola, possibly due to the tissue distortion produced in this region.

**Fig 1:**
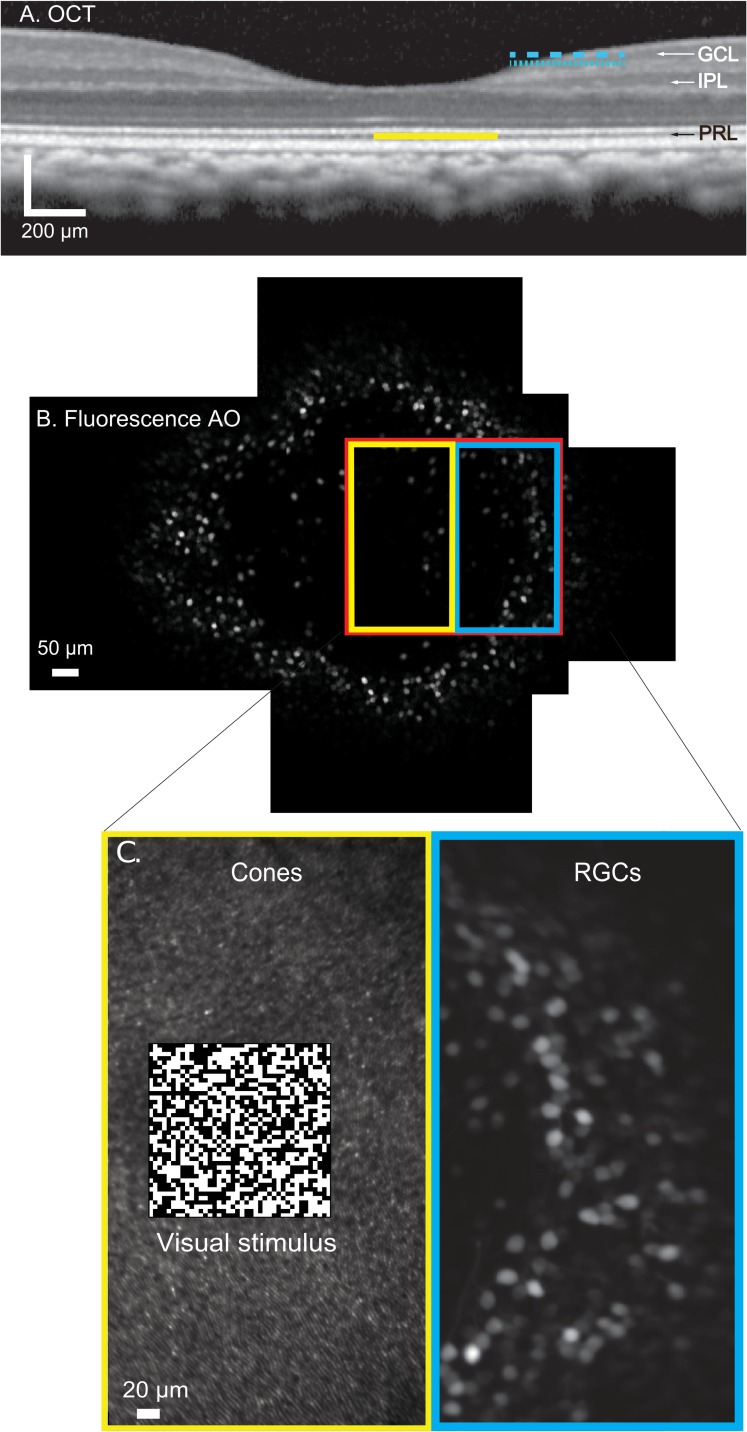
Illustration of the method. **a)** Optical coherence tomography (OCT) of the fovea of the imaged monkey. The response of G-CaMP6s expressing retinal ganglion cells, located in the ganglion cell layer (GCL), were recorded with a 488 nm laser focused at two depths within the GCL (dotted and dashed blue lines). Cone photoreceptors located in the center of the fovea were stimulated with a 561 nm laser focused at the photoreceptor layer (PRL) (yellow). Simultaneously cone photoreceptors were imaged in reflectance using a 796nm laser, also focused at the PRL reflectance to provide a high signal/noise image for both registering the fluorescent images of the RGCs and stabilizing the checkerboard stimulus on the moving retina. **b.)** Montage of high-resolution fluorescent adaptive optics (AO) images showing G-CaMP expression in the foveal ring of RGCs imaged in this study. Yellow, blue and red rectangles show the arrangement of the fluorescence imaging field, the visual stimulation, and reflectance imaging regions respectively. **c.)** Expanded schematic of the stimulation and recording paradigm. The yellow region contains an AO reflectance image of photoreceptors in the stimulation field and the stabilized visual stimulus consisting of a 40 x 40 check binary white noise checkerboard presented to the foveolar cones. The blue region contains an AO fluorescence image of RGCs recorded from. In subsequent conditions the right-left configuration of the stimulation and imaging fields was rotated to image the temporal, superior and inferior portions of the foveal ring.

This manuscript reports the functional mapping between foveolar RGCs and their receptive fields, produced by a non-invasive approach that can locate hundreds of foveal RGC receptive fields simultaneously *in vivo* (Fig 1). By combining cellular scale adaptive optics imaging of macaque retina expressing the calcium indicator GCaMP6s with the presentation of high precision, high spatial frequency binary white noise visual stimuli, we have revealed the functional topography of the foveola of a living primate.

## Results

### A functional map of the primate foveola

**Fig 2:**
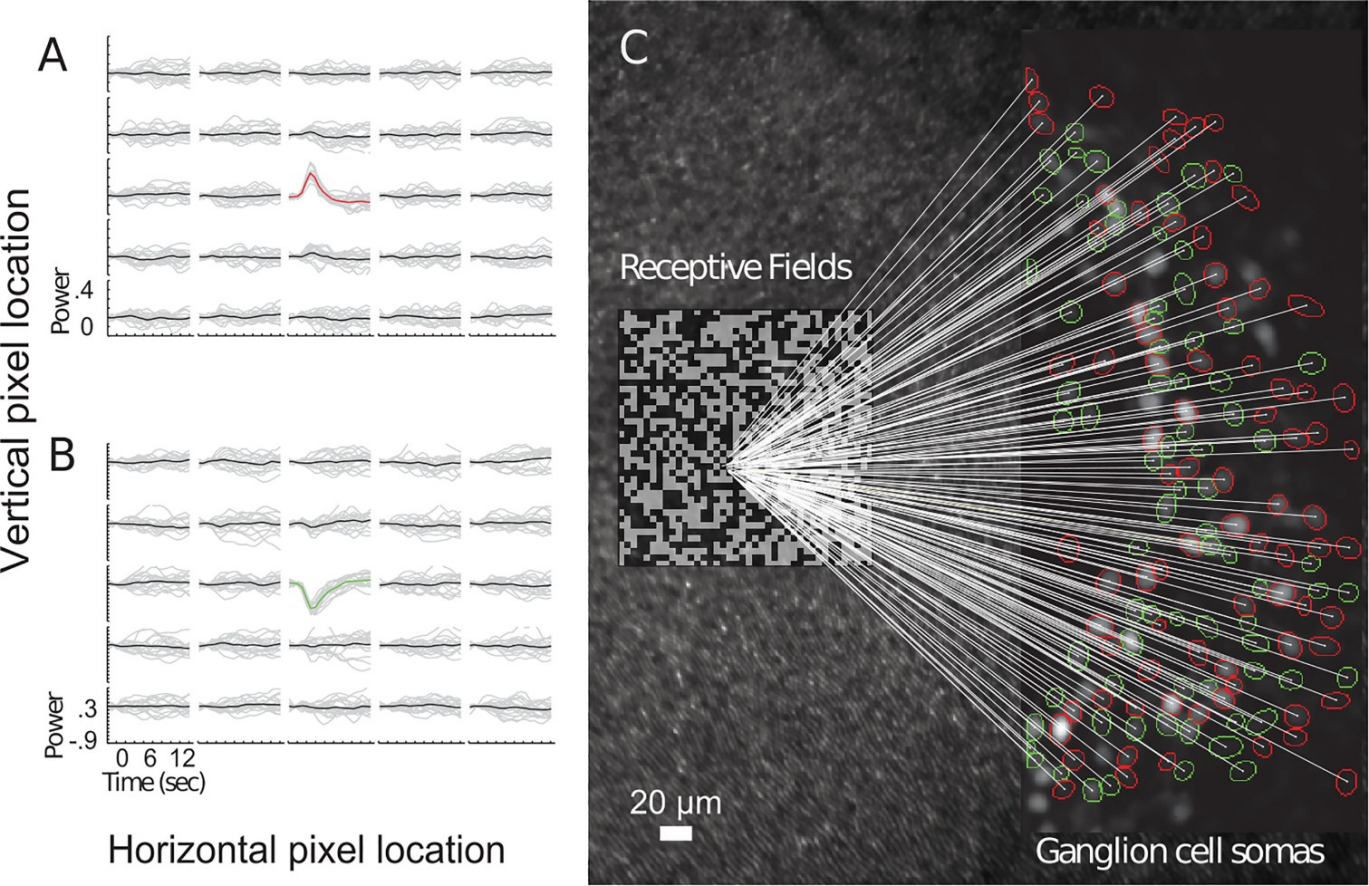
Mapping GC impulse responses. **a.) and b.).** Two examples illustrating the method of calculating the spatio-temporal response to individual checks of the stimulus. These images show the temporal response to light in a 5 by 5 array of checks centered on the check that resulted in the maximal response. A positive (ON-dominant) response is seen in Fig 2A) and a negative (OFF-dominant) response in Fig 2B). In each plot, grey traces show individual responses to 10 presentations of the check, whereas dark lines show mean response to the 10 presentations, with non-significant responses shown as black and significant responses shown as red (ON-dominant) or green (OFF-dominant). **c.)** Projection from centroids of receptive fields (RF) near the fovea center to RGCs measured using white-noise analysis. The background of the figure shows cones imaged by reflectance imaging across the entire AO field. All RGCs recorded in this example data set are on the temporal side of the fovea. The location of the checkerboard stimulus illustrated in Fig 1 is shown to the center left of the figure, covering the center of the fovea. Subsequent datasets were recorded RGCs nasally, inferior and superior to the fovea, and the complete data set is shown in Fig 3. Straight lines connect the pixel at the center of the RF to the RGC activated. The polarity of each RGC is indicated by the color of the contour around its soma (red, ON; green; OFF).

To produce a high-resolution functional wiring diagram of the foveola, we imaged RGC impulse responses following the presentation of randomized check stimuli on the scale of a few foveal cones (Fig 2). Only statistically significant impulse responses were mapped (see Methods). RGC receptive fields were classified as ‘ON-dominant’, those showing a positive correlation between visual stimulus intensity and the RGC fluorescence measured 1–2 seconds after the visual stimulus change, and ‘OFF-dominant’, those showing a negative correlation (Fig 2). An approximately equal proportion of ON-dominant and OFF-dominant RGCs was observed in all areas except the nasal fovea where ON-dominant cells were more numerous, particularly in the superficial layer of ganglion cells where ON-dominant RGCs outnumbered OFF-dominant five-fold (S2 Table).

**Fig 3:**
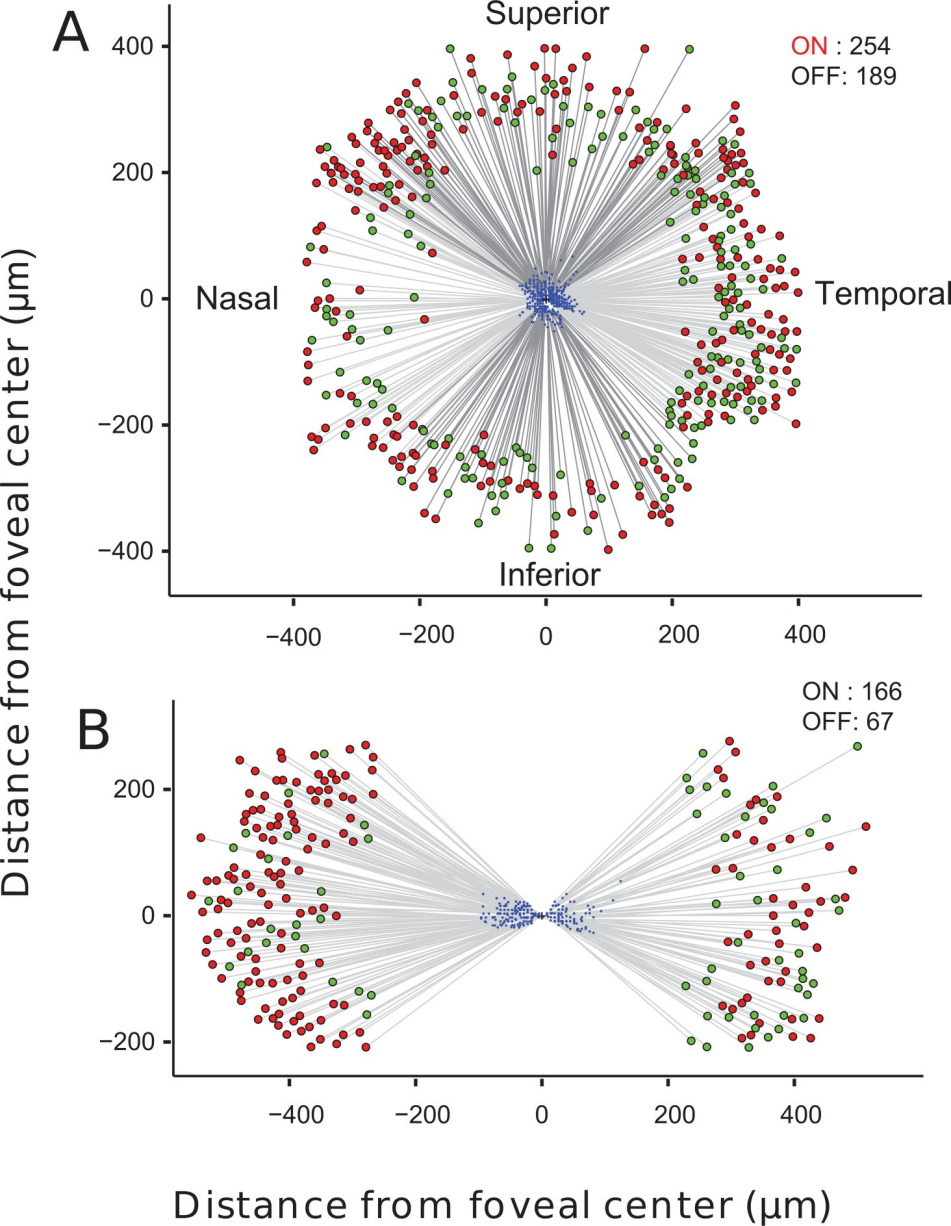
Combined foveolar GC receptive field maps for superficial and deep recording positions. ON cells are represented by red circles and OFF cells by green circles. **a.)** Projections to RGCs deep in the GCL, near the outer plexiform layer (OPL) in temporal, nasal, superior and inferior azimuths. Straight lines connect the pixel at the center of the RF to the RGC activated. **b.)** Nasal and Temporal projections to RGCs located more superficially in the GCL than those shown in Fig 4A. RGC somas at the more superficial depth extend to greater eccentricities from the fovea center and the receptive fields of these cells also cover a wider range of eccentricities.

Receptive field mapping was performed for the superior, inferior, nasal and temporal portions of the retinal ganglion cell ring, at two focal depths. Fig 3 shows a spatial map of receptive fields and the centroids of the foveal RGC cell somas to which they are functionally connected for the whole foveal ring. This data revealed how the position of RGC somas in the ring maps onto the receptive field location. This information is crucial for vision restoration therapies that rely on bypassing degenerated photoreceptors and directly stimulating the RGC somas themselves. If RGC soma positions were scrambled relative to the arrangement of their receptive field locations, it would be challenging to create an RGC stimulation pattern that would mimic visual stimulation at the fovea. Additionally these data allowed us to identify the ‘physiological center’ of the fovea, the location where neighboring receptive fields project to RGC somas on opposite sides of the ring. This position is marked by a cross at the origin of Fig 3.

### Quantifying the retinotopic organization of RGCs

**Fig 4:**
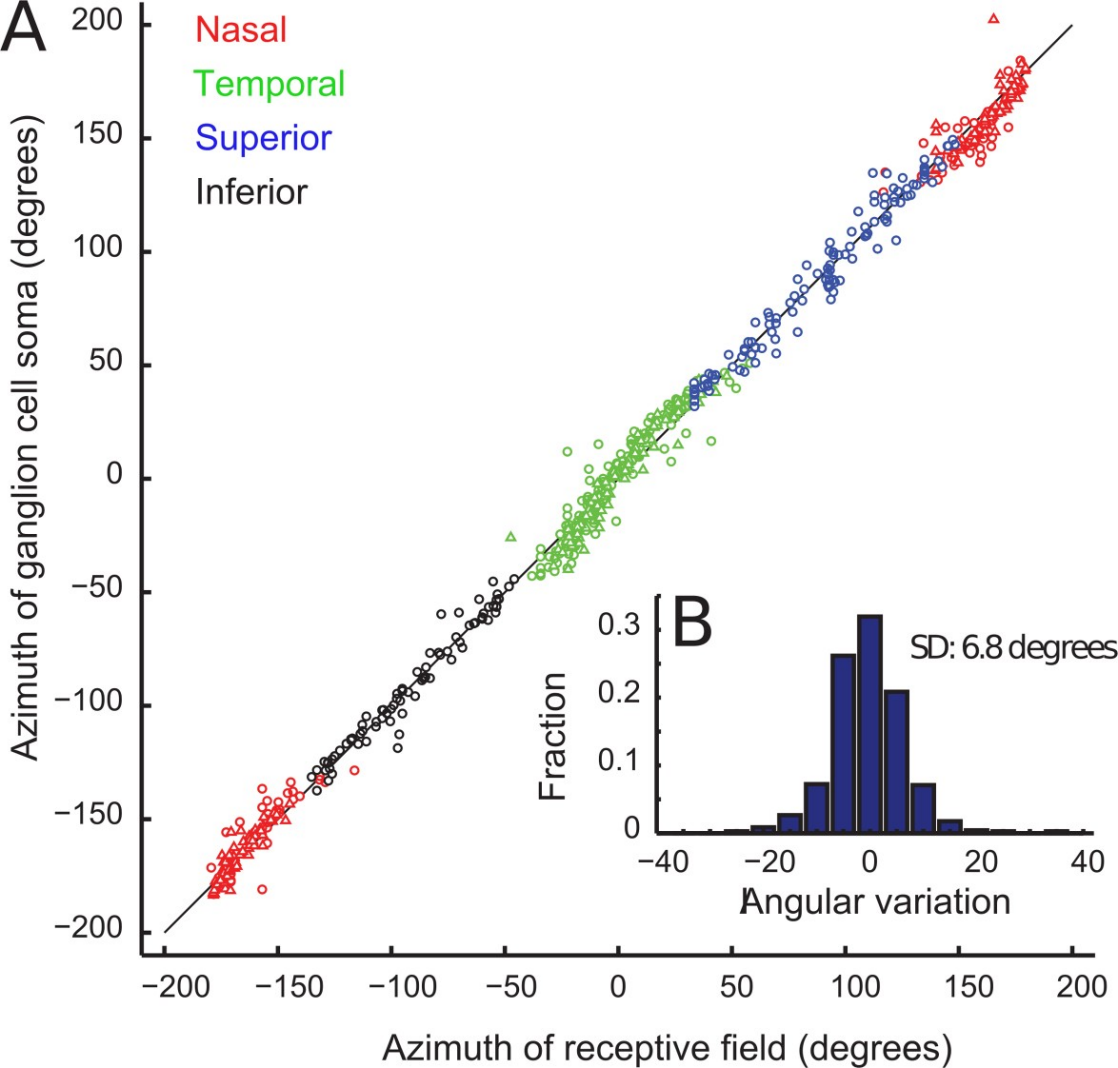
Preservation of receptive field azimuth in the location of GCs in the dilated foveal ring. **a.)** Biplot of the azimuth of individual retinal ganglion cells versus the azimuth of the measured receptive field of each ganglion cell. Color of symbols show results for nasal, temporal, superior and inferior directions from the fovea center. **b.)** Distribution of the angular difference between azimuths of GC somas and the azimuth of the receptive fields, centered around 0.38 degrees and with a standard deviation of 6.8 deg.

To quantify how precisely the geometry of foveolar receptive fields is preserved in the spatial arrangement of RGC somas, we first examined the azimuthal data. The orderly displacement of RGC somas relative to the cones that serve them is clear in the linear relationship between the azimuthal angle of the receptive field and the corresponding azimuthal angle of the radially displaced ganglion cell soma in both superficial and deep layers (Fig 4). The angle between pairs of vectors connecting the physiological foveal center (the origin in Fig 3) to each receptive field and the corresponding vector from the origin to the RGC soma centroid position associated with that receptive field, were normally distributed around 0.38 (Kolmogorov-Smirnov-test, N = 676, p = 0.286, KS statistic 0.53) with a standard deviation of 6.8 degrees.

**Fig 5:**
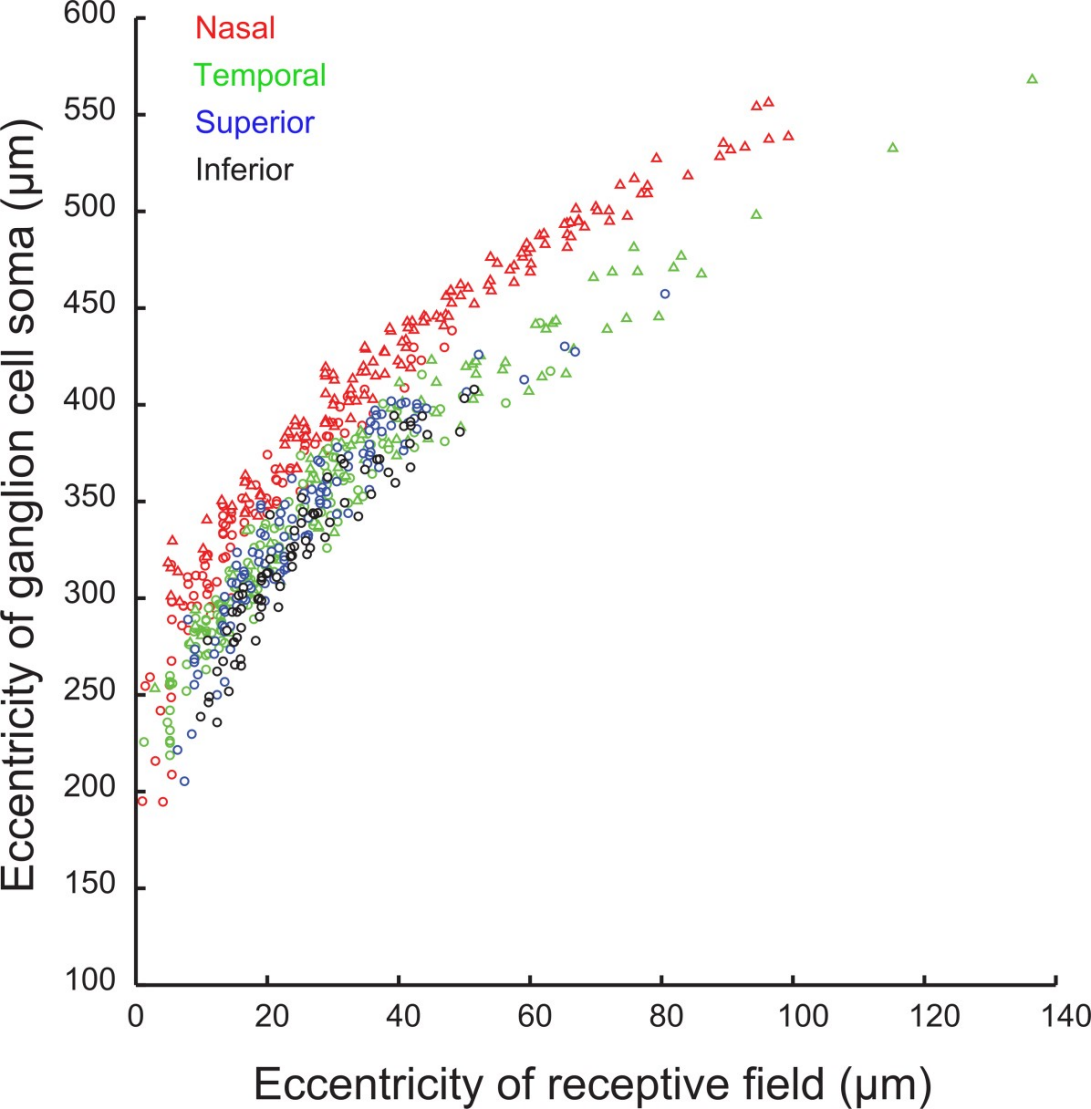
Relation of the eccentricity of individual retinal ganglion cells to the eccentricity of their receptive fields. Circles represent data point taken at the deep focal position, triangles represent data taken at a superficial focal position.

The eccentricity of each ganglion cell soma relative to its receptive field location is presented in Fig 5. The distance between the most central receptive fields and their RGC somas is approximately 220 μm. The distance between the receptive field and the corresponding RGC increases with increasing distance from the foveal center and is greater on the nasal side of the retina. 40 μm from the foveal center the receptive field to RGC displacement had grown to 386±29 (95% confidence interval) μm nasally and 348±24 (95% confidence interval) μm temporally. Polynomial fits and confidence intervals for all our data are shown in S1 Fig with corresponding fit coefficients listed in S1 Table.

### Relating the functional geometry of RGCs to cone density

**Fig 6:**
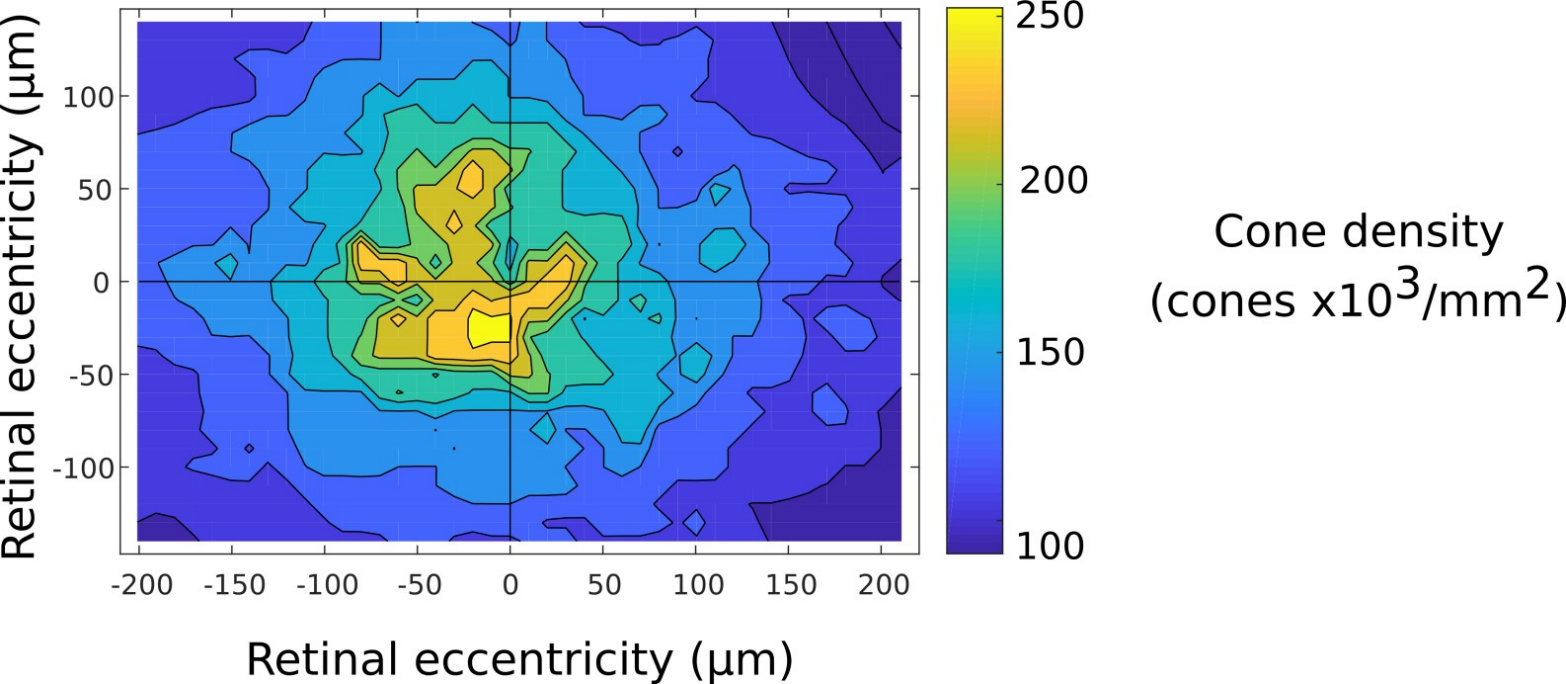
Cone isodensity plot for the same eye used for functional recording. Cone densities calculated using automatic cone identification [18] and nearest neighbor distances. The contour plot was generated by interpolating between averages of 30 μm x 30 μm squares sampled every 10 μm in a square array. Contours are separated by 18,200 cones per mm^2^. The physiological foveal center is located at the origin with nasal and inferior directions corresponding to negative numbers. The majority of physiological recordings were made in a 200 μm x 200 μm region centered on the origin.

To test the hypothesis that RGC eccentricity is linked to cone density [7], we mapped foveolar cone density in the same animal. A contour plot of cone density in the near foveal region is presented in Fig 6. The density of cones in a 100 μm x100 μm region to the nasal side of the physiological foveal center was significantly higher than the temporal side (Wilcoxon rank sum, p = 10^−14^) and the region of highest median cone density (253,000± 7000 (SD) cones mm^2^) did not coincide with the physiologiological center based on ganglion cell projections, which had a median density of only 186,000±22,000 (SD) cones mm^2^. Peak cone density was displaced 29μm naso-inferiorly with respect to the physiological foveal center.

**Fig 7:**
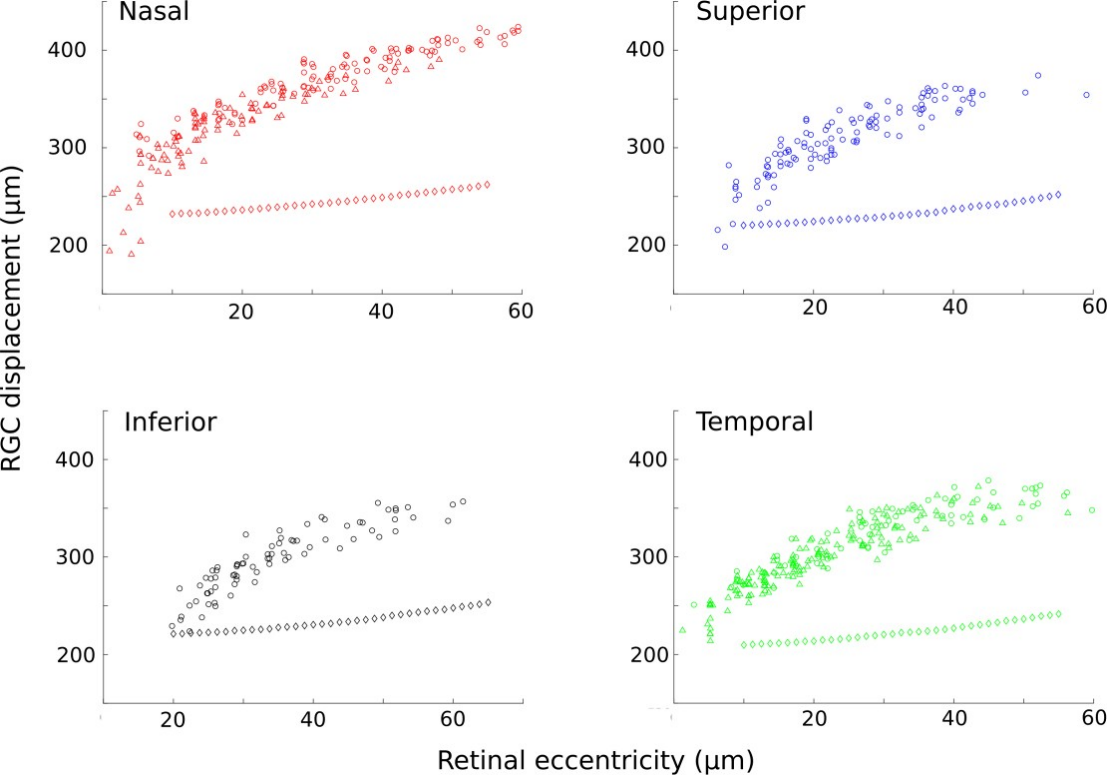
Comparison of measured and predicted RGC displacements as a function of receptive field eccentricity for each of the 4 quadrants of the foveola. Triangles represent data taken at a superficial focus, circles represent data taken at the deeper focal position (see online methods). Diamonds represent predicted RGC displacements based on a geometrical model for cone pedicle displacement proposed by Schein [7].

The displacement between the receptive field position and the RGC soma is graphed separately for each of the four foveal quadrants in Fig 7. These data are compared to predicted values based on the cone density distribution in that quadrant and a model of foveal development proposed by Schein [7]. We show that for receptive field eccentricities under 70 μm, RGC displacement increases more rapidly as a function of receptive field position, than Schein’s purely geometrical model predicts.

### High magnification functional imaging

**Fig 8:**
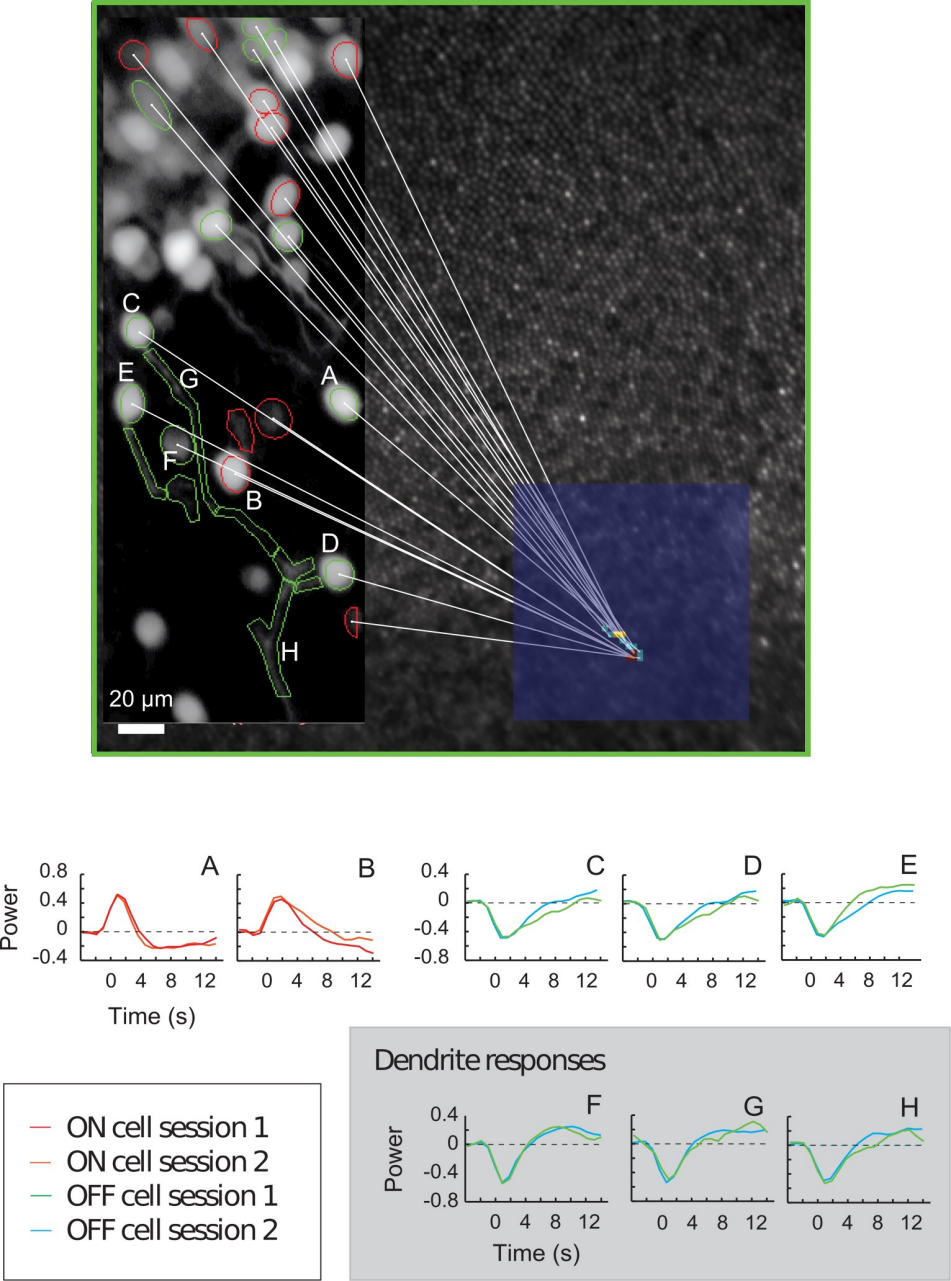
Consistency of receptive field mapping across sessions. **A**. Map of the locations of cells and dendrites whose consistency of response is shown in Fig 8B. **B**. Temporal impulse response of retinal ON dominant RGCs (A and B) OFF dominant RGCs (C, D and E) and dendrites (F,G and H) on two imaging sessions separated by one week.

Ganglion cells in the inner-most portion of the foveal ring receive inputs from cones in the most central part of the fovea, as shown in Fig 4. Using a smaller imaging field, in which individual stimulus checks approached the size of foveal cones (Fig 8), we recorded impulse responses not only from ganglion cell somas, but also from dendrites responding to the same stimulus. While this reduced field of view decreased the number of RGCs imaged, it allowed us to directly compare the properties of ganglion cell somas and dendrites across sessions and verify the consistency of our methods. Fig 8 shows data from RGC somas and dendrite groups imaged one week apart; reproducible impulse responses to stimulation by the same check are observed.

## Discussion

Building on methods introduced by Yin et al., [19] we have extracted precise topographic information about retinal structure coincident with *in vivo* recording from hundreds of retinal ganglion cells in an intact macaque. The high spatial resolution of the adaptive optics system allowed us to both image responsive retinal ganglion cells and to provide focal stimulation on the spatial scale of a few foveal cones. This approach enabled consistent functional imaging of hundreds of individual retinal ganglion cells serving the most central, formerly inaccessible foveal cones. We observe a precise retinotopic mapping from receptive field locations to their displaced ganglion cell somas despite the multiple developmental processes which give rise to fovea.

### RGCs have a precise retinotopic organization

The inward migration of cones during development and the opposing outward movement of ganglion cells results in an adult retina in which ganglion cells are displaced radially from their receptive fields [3,20,21]. The present findings reveal the precision with which foveal topography is maintained during these developmental processes. We do not find evidence of potential crossing of Henle fibers that could have been introduced if there were radial or tangential migration of photoreceptors during development. This striking topographic regularity of ganglion cells could be a passive consequence of developmental forces in the formation of the fovea, but it may also increase the efficiency with which cortical circuits can be wired that favor local connectivity [22].

### Nasal temporal asymmetry is present even at the lowest eccentricities

RGC displacement is understood to increase to a maximum at around 0.6 mm in humans [5], declining again at larger eccentricities. Few studies have collected data at eccentricities below the peak and our data is the first to report RGC displacements from receptive fields under 100 μm from the foveal center. The spatial extent of GCaMP expression permits recordings from RCGs up to 1.5 mm [23] from the center of the fovea but in this case our observations extend to 0.76 mm based on the maximum scan angle of our imaging system. This data covers the central most fovea, where the separation between RGCs and their receptive fields is yet to reach its maximum. Our *in vivo* measurements are unusual in that unlike histology they are not subject to preparation artifacts or the challenges of sectional reconstruction.

RGC’s serving the most central receptive fields at zero eccentricity show displacements of approximately 220μm. Receptive fields located 40 μm from the foveal center have RGC displacements of 386 ± 29 μm nasally and 348 ± 24 μm temporally. This is the first data recorded at these low eccentricities and the displacement length is substantially longer than modeled values reported for the human retina [5] which, rescaled based on the axial length of the smaller macaque eye would correspond to 100 μm at an eccentricity of 40 μm. Drasdo *et al*.,[5] (human) and Perry and Cowey [6] (macaque) estimate the post-receptoral displacement to be 20% and 15% of the total, which, applied to our data, produces an estimated length of a Henle fiber of approximately 180 μm for the most central receptive fields, and 330 μm nasally and 300 μm temporally at 40 μm.

These results may indicate that RGC displacement rises more steeply as a function of eccentricity than previously assumed, or this may reflect individual differences amongst the foveal architecture of macaques. In humans an 8 fold variation in the area of the foveal avascular zone [24] has been reported, therefore variation of this magnitude in RGC displacement is certainly plausible in macaque.

The nasal–temporal asymmetry in RGC displacement observed previously in humans and primates [5,6] was also clear in our data. This difference may reflect the greater cone density in the nasal hemiretina, consistent with Schein’s view [7] that the radial displacement of cone pedicles relative to cone inner segments is just sufficient to match the greater area required by the larger number of cone pedicles.

We explored this hypothesis further using Schein’s geometrical model to predict Henle fiber length based on the measured cone density distribution in this animal. Converting these predictions to comparable RGC displacements using Schein’s data on post-receptoral displacement indicated that for receptive field locations under 70 μm from the foveal center, RGC displacement is not well described by the model. Displacements increase more rapidly than predicted despite good agreement with cone densities in the original model. This difference could be attributed to cone pedicles at very low eccentricities being larger than assumed or alternatively it may be that while packing density drives Henle fiber length at larger eccentricities, the displacement of the RGCs that serve receptive fields in the foveola is not governed by geometrical constraints but rather is determined by developmental cues that drive RGC migration in this most privileged and anatomically unusual region of the eye. Recent AO-OCT data [25] measuring ganglion cell density in the living human shows lower ganglion cell densities compared to histological estimates, which is consistent with our finding of longer Henle fibers.

### Defining the ‘physiological center’ of the fovea

Reporting the location of ‘the fovea center’ has been a vexing question as the preferred retinal locus of fixation does not correspond in any systematic way to peak cone density, the center of the avascular zone or the bottom of the foveal pit [24,26]. Understanding these relationships may provide insight into the developmental processes that govern the formation of the fovea. Here we identify a novel definition of the foveal center based on functional connectivity, a location that is of particular developmental significance as it marks the boundary of the two hemifields. Early in development ganglion cells secrete factors to trigger the creation of an avascular zone [3], it would be intriguing to know if this physiological center corresponds to the center of the avascular zone or perhaps the preferred retinal locus of fixation itself.

### Implications for vision restoration

Our results have important implications for attempts to restore foveal vision in outer retinal degeneration by rendering the ganglion cells light sensitive with optogenetic methods. The dramatic dilation of ganglion cell position with respect to their respective fields creates the problem of potential gross perceptual distortion of the retinal image. Psychophysical experiments testing spatial vision following retinal distortion make it unlikely that the brain could learn to compensate for this distortion, favoring an alternative restoration approach in which vision is restored by reactivating residual cones [27] rather than ganglion cells. However, the strict retinotopic organization of ganglion cells we report here makes it possible that, with improvements in head-mounted eye tracking technology, one could stabilize a predistorted retinal image that compensates for ganglion cell displacement from the cones that drive them.

### Future improvements

Despite the ability to record from large numbers of cells in the living eye, the GCaMP method remains vulnerable to possible sources of sampling bias. Expression is confined to a peri-foveal ring and the duration and level of expression of GCaMP in primate may be variable between individuals. In this study, large number of RGCs that expressed GCaMP did not show a statistically significant impulse response (S2 Table). These cells may have been unresponsive due to high levels of G-CaMP expression or may not have been optimally driven by the stimulus. We observed an inverse relationship between the brightness of a cell and the speed and amplitude of the response, suggesting that high levels of G-CaMP expression may compromise RGCs normal physiological function (S2 Fig). Some cells stopped fluorescing entirely, potentially due to GCaMP toxicity. Cell loss due to G-CaMP expression has been documented [28,29] but there is promise that use of the TET-On method [30] to reduce G-CaMP expression at times when imaging is not underway will minimize this challenge.

In subsequent studies it may be desirable to produce the equivalent of a spike triggered average map of receptive field of each RGC, however the number of stimulus presentations in the white noise sequence is limited by the slow temporal dynamics of the GCaMP mediated calcium response and the signal from each presentation is limited by light safety considerations. Future studies may aggregate data over multiple days or design more specific stimuli to characterize larger receptive fields and more complex RGC properties like direction or vergence selectivity. With dedicated stimuli targeting single cones and high frequency eye tracking with shorter stimulus stabilization times similar to those used in AOSLO psychophysics experiments in humans [31–33] and recordings from macaque LGN [34], we may be able to further elucidate the fine structure of the smallest, most central foveal GC receptive fields.

## Methods

### Animal care

The macaques were pair housed in an AAALAC accredited vivarium, fed ad libitum with a nutritious lab chow, supplemented with dried treats and fruits and vegetables such as mangos and carrots, and were given a novel enrichment item once a week such as browse (leaf covered tree branches), raisin stuffed pinecones and treat filled forage boxes. Daily primate enrichment included 2 pieces of manipulata, puzzle feeders rotated among all animals, weekly videos or music and rotating access to a large, free ranging space with swings, perches, etc. They were cared for by Division of Comparative Medicine veterinary staff under the supervision of four full-time veterinarians, two with residence training in primatology, as well as 6 veterinary technicians, who monitor the health of the monkeys and check for signs of discomfort at least twice daily. This study was carried out in strict accordance with the ARVO Statement for the Use of Animals and the recommendations in the Guide for the Care and Use of Laboratory Animals of the National Institutes of Health. The protocol was approved by the University Committee on Animal Resources of the University of Rochester (PHS assurance number: D16-00188(A3292-01). The animal used in this study was returned to the colony after these experiments were concluded and transferred to another project.

### AAV mediated gene delivery to retina

G-CaMP6s[35] was expressed in neurons of the inner retina by the intravitreal injection of 100 ul of AAV9-CAG-G-CaMP6s (vector AV-9-PV2833 from the Penn viral vector core). A three year old male *Macacus fascicularis* was injected intravitreally in the left eye with 100 ul of AAV9-CAG-G-CaMP6s as described previously [19]. Previous measurement of antibody titer in this monkey established that there were no detectable (neutralizing titer less than 1:10) inactivating antibodies against AAV9. 50 ul intravitreal injections of triamcinolone acetate (Kenalog, Bristol-Myers Squibb) were administered every 4–5 weeks as needed to minimize retinal inflammation. On the few occasions when slight vascular darkening was observed, it was eliminated by this treatment in approximately 10 days.

### Assessment of G-CaMP6s expression

Expression was tracked by fluorescence fundus imaging with a 488nm confocal scanning laser ophthalmoscope (Spectralis, Heidelberg Engineering). High levels of G-CaMP6s expression were present in a ring around the fovea when first examined at 3.5 weeks post injection. The G-CaMP signal diminished slightly over time and some previously labeled cells no longer expressed G-CaMP (S2 File and S3 Fig). The data presented here was collected over a period of 6 months, beginning 8 months after injection of the viral vector.

### Anesthesia and animal preparation

The macaque was intubated and anesthetized with isoflurane, the concentration of which was adjusted to effect (1–3%). Paralysis was induced with intravenous vecuronium bromide. During anesthesia, heart rate, electrocardiogram, blood oxygenation and respiratory rate were continuously monitored. A rigid gas permeable contact lens (11mm diameter, 5.8mm base curve, -2.0D) was fitted to protect the cornea, and correct spherical aberration of the imaged eye. Pupils were dilated with 1–2 drops of phenylephrine hydrochloride (2.5%) and Tropicamide (1%). The macaque’s head was held steady with a head post attached to a positioning system that rotated around the nodal point of the eye as described in Yin *et al*., 2014 [19]. Imaging took place no more than once per week.

### Adaptive optics (AO) imaging and visual stimulation

All imaging was performed with the single photon primate fluorescence AOSLO at the University of Rochester. The imaging portion of the system is described in detail in Gray *et al*., 2008 [36] and Yin *et al*., 2014 [19]. Scan angles of the system were calibrated with a model eye using grids of known size and for all images of the macaque eye a scaling value of 223μm/degree was applied. The mean luminances of the 561nm visual stimulus light and the 488nm imaging light were 1.7 μWcm^-2^ and 55.8 μWcm^-2^ respectively.

Fig 1A shows an OCT image of the fovea of the imaged monkey. The blue dashed and dotted lines illustrate the approximate planes of focus of the 488nm laser used to excite G-CaMP fluorescence emitted by RGC. These two focus depths, separated by 0.34D, were used to image RGCs superficial and deeper within the ganglion cell layer. Fluorescence emission was collected by a PMT with a 3 Airy disk diameter pinhole and 520/35nm bandpass filter in the detection path. The yellow line illustrates the plane of simultaneous 561 nm visual stimulation and 796 nm reflectance imaging field corresponding to the plane of the central foveal cones.

Fig 1B shows an en face fluorescence AOSLO montage of the foveal region imaged in this study. A 3.4 x 2.5 deg (6.1 μm per check) scan field was used to image superficial RGCs, this could be reduced to 2.3 x 2.5 (4μm) to image at the deeper focal position. The accessible scan area (red rectangle) was split into areas of visual stimulation (yellow rectangle) and imaging (blue rectangle) as shown in Fig 1B. The scan field was placed on the nasal portion of the central fovea and ring of G-CaMP expressing RGCs, and subsequent recording sessions placed similar imaging and stimulation fields on temporal, superior and inferior fovea. In later sessions, to obtain higher resolution data on cone to ganglion cell projections and assess the consistency of responses, the entire field was reduced in size to 1.3 x 1.3 deg, reducing the size of checks to 2.6μm, a lengthscale slightly larger than the 2.25 μm diameter of the smallest foveal cones measured in this study. All scan field and stimulation parameters are summarized in S3 Table.

The 40 x 40 check stimulus was centered on the fovea, with each check corresponding to 4x4 pixels of the imaging field. The precision of stimulus stabilization was 1 pixel r.m.s. corresponding to 1.55μm (0.4 minutes of arc), 1μm (0.25 minutes of arc) or 0.65μm (0.15 minutes of arc) depending on which field of view size was used. This precision could be maintained over a period of approximately 200 seconds and also afforded the ability to aggregate data from repeat trials at the same location (for details of the stabilization approach, see Supplementary materials S1 File and S4 Fig). The relative position of the receptive field and ganglion cell centroids is not affected by stimulus placement uncertainty as the imaging and stimulation fields are locked together, therefore the precision of this measurement is higher, at 0.5 pixels corresponding to 0.78, 0.5 or 0.33μm depending on the field of view. Individual checks were pseudo-randomly assigned to be black or white (100% contrast) at 0.5 Hz, matching the slow kinetics of the RGC calcium responses. This visual stimulus was generated by digital modulation of the stimulus laser, which produces higher contrast between maximum and minimum intensity (>1.5 log units) than the acousto-optic-modulator (AOM) used in earlier studies[19].

### Data analysis

Reflectance and fluorescence images were acquired at a rate of 26.5 Hz for the duration of each trial and desinusoided in real time [32] to remove image distortion caused by the resonant scanner. Fluorescence image stacks were co-registered with the simultaneously acquired reflectance image stacks to compensate for eye movements and then summed to generate single images as described in Yin *et al*., 2014 [19]. The location of individual ganglion cell bodies was segmented automatically using ‘Two Photon Processor [37]’ a freely available software package for MATLAB, then manually inspected and modified to an ellipsoidal shape using ImageJ [38].

The fluorescence intensity for each cell was normalized with respect to a ‘baseline’ calculated as the mean fluorescence intensity within a two second time window before the stimulus onset. For each RGC we calculated the temporal impulse response to each check by reverse correlating [39,40] check contrast with normalized peak fluorescence intensity of the RGC for at least 10 check presentations. Mean responses for each cell were then calculated for each check and two inclusion criteria were imposed to determine if the mean response to any particular check was significantly different from chance (see statistics section). The total spatial impulse response for each cell was calculated as the combined weighted mean of those significant responses to individual checks that increased the signal to noise ratio of the overall response. This process identified the checks corresponding to the receptive field for that cell. This final step is similar to the more common ‘spike triggered average’ but could instead be considered an ‘impulse response triggered average’. Only rarely was more than one check identified. This analysis allowed us to identify the location of the estimated receptive field centroid. Based on the estimated temporal impulse of the RF center (Fig 2), ganglion cells could be classified as ON-center or OFF-dominant types.

### Calculating the foveal center

The physiological foveal center was calculated as the convergent location of lines from each RGC through its receptive field for each field of view. This was computed by minimizing the absolute angular difference between pairs of vectors connecting this point with either each RF and the corresponding RGC soma Convergence of the nasal and temporal data (as shown in Fig 3 (superficial)) to the same retinal location suggested that this approach was appropriate. Combining data across multiple imaging sessions required correcting for any shift in absolute location due to chromatic aberration, therefore data from images acquired on separate days was aligned using the calculated foveal center for that imaging session.

### Cone density

Cone densities were calculated using a 796nm reflectance image montage of the fovea of the same macaque. Cones were identified automatically [18] and the nearest neighbor distances calculated, from which densities could be computed by assuming a hexagonal cone array. To generate a low noise contour plot, the montaged image was divided into smaller regions of interest (ROIs) 30x30 microns in size. Centers of ROIs were spaced 10 microns apart in a square lattice such that ROI areas overlapped. Cones whose computed centers were within two pixels of the edge of the ROI were discarded based on the assumption that 2 pixels was the approximate radius of a cone. The median nearest neighbour distance was considered representative of the distribution (tailed, non-normal) within the ROI and this value was assigned to the center location of that ROI. These data were interpolated to produce contour plots of cone density.

### Statistics

The inclusion procedure for identifying significant RGC responses had two steps. The first was to determine if the peak response to any particular check was more than 3.5 times the standard deviation of the response to all checks. Potentially significant responses were then subject to a paired T-test α = 0.05 comparing the peak response to the baseline normalized fluorescence intensity for that particular cell. Only cell responses that met both of these criteria were included in further analysis. The number of cells identified as unresponsive to any check together with the total number of cells analyzed is shown in S2 Table.

A two-sample Kolmogorov-Smirnov (KS) test was used to determine whether the azimuthal difference between the vector from the foveal center to the centroid of the receptive field and the vector from the center to the ganglion cell soma were normally distributed around zero. Failure to reject the null hypothesis (p>0.05) means the azimuths are drawn from the same continuous distribution. The MATLAB function kstest2() was used. The azimuthal difference between the receptive field vector and its corresponding ganglion cell vector was determined for each pair. The mean difference and standard deviation were calculated.

The distribution of nearest neighbour distances between cones was tailed and non-normal, as there was a lower bound placed on the inter cone distance to prevent the detection of multiple peaks within the same cone. The median was therefore chosen as the most appropriate representative value for this distribution. Standard deviation for each median value of cone density was computed using a bootstrapping method with one thousand resamples using the MATLAB function bootstrp(). To test whether the nasal fovea had a higher cone density, a Wilcoxon rank sum test [41] (MATLAB function ranksum())was performed on raw nearest neighbour distances within two 100μm x 100μm squares, one nasal (n = 721) and one temporal (n = 695) to the computed foveal center.

### Modeling RGC displacement as a function of receptive field position

A model predicting Henle fiber length based on geometrical constraints arising from cone outer segment and pedicle density was proposed by Schein [7]. We adapted Schein’s model which relied on histological data and assumed radial symmetry, to incorporate our *in vivo* data on the cone density distribution. An azimuthally symmetric linear model of cone pedicle density was assumed from Schein’s data on pedicle density close to the fovea as ρ = 18.825 + 8.42*r where r is the anatomical radius from the center of the fovea. Assuming that each foveal cone has one pedicle, then the number of cones N in an area must correspond to the same number of pedicles within a given annulus of known inner radius r_0_ and unknown outer radius r_p_. The inner radius corresponds to the distance at which the first pedicles from cones at the center of the fovea are located. Thus, $N=\int_{r_{0}}^{r_{p}}\;2\pi \;\rho \;r\;dr$. Using the linear model from above, this becomes a cubic equation that can be solved analytically for r_p_, given a known N and r_0_.

To determine N(r) in this animal, cones in a 90 degree wedge of radius r_c_ extending out from the fovea were counted in MATLAB using the method [18] described earlier. Estimating the post receptoral distance as 15% of the total RGC displacement in macaque [5], a value of 187 microns was estimated using the RGC displacements of the most central receptive fields measured in this study. Using the cubic formula, the outer radius of the area containing pedicles connected to the counted cones was calculated. This process was then repeated for the same angular wedge, but at increasing radii, to build a profile of r_p_ as a function of r_c_. The process was repeated for all four wedges to graph the nasal, temporal, superior, and inferior regions of the fovea out to an eccentricity of 70μm. To verify that our model was working as intended we recapitulated the figures produced in Stein^7^ using his radially symmetric model for cone density, we note that our measured cone densities were within 10% of those assumed by Schein, well within inter-subject variability.

To allow direct comparison between the modeled pedicle positions to the RGC displacements measured in this study pedicle positions were divided by 0.85 based on histological estimates of post-receptoral distances in the macaque [5]. Fig 7 shows RGC displacement as a function of receptive field eccentricity compared to predicted RCG displacements based on the model developed by Schein [7]. Modeled RCG displacements were calculated by subtracting r_c_ from r_p_ and dividing by 0.85_._ Measured displacements were calculated by subtracting receptive field eccentricity from RGC soma eccentricity (centroid). Modelling starts at 10 μm for each quadrant as below this value there were less than 5 cones, which was too small a sample size to reliably calculate a meaningful cone density.

### Light safety

The total light exposure for retinal locations over the entire imaging session was calculated, taking into account all imaging lights used (488 nm at 10uW, 796 nm at 200uW, and 843 nm at 30uW), and the light used for visual stimulation (561 nm at 0.6uW maximum). Total light exposure at any retinal location did not exceed the American National Standards Institute (American National Standard for the Safe Use of Lasers ANSI Z136.1–2007) maximum permissible exposure for human retina [42] scaled by the ratio of the numerical aperture (NA) of human to non-human primate eyes (NA_Human_^2^/NA_Non-human primate_^2^ = 0.78)[43]. Because the minimal interval between imaging sessions was at least 5 days, accumulation of exposure over multiple sessions was not considered. Power levels were measured at the pupil plane immediately prior to each imaging session.

## Supporting information

S1 File Real time stabilization of the visual stimulus on retina. (DOCX)

S2 File Long-term stability of GCaMP expression. (DOCX)

S1 Table Coefficients and 95% confidence intervals for least squares fits of 5^th^ order polynomial to RGC soma displacement as a function of receptive field eccentricity. (DOCX)

S2 Table Numbers of ON- or OFF- dominant ganglion cells measured at different locations in fovea. (DOCX)

S3 Table Configuration of imaging field and spatial resolution of visual stimulus. (DOCX)

S1 Fig Displacement of RGC soma relative to eccentricity of receptive field for nasal, temporal, inferior and superior quadrants. Triangles represent data taken at a superficial focus, circles represent data taken at the deeper focal position (see methods). Data were fitted with a 5^th^ order polynomial, 95% confidence intervals are indicated by dashed lines.
(TIF)

S2 Fig Time course of the response of foveal RGCs to stimulation by the most effective check. A. Impulse response of 40 foveal RGCs sorted into red ON responses (positive response to light stimulation) and green OFF responses (negative response to stimulation).
B. Impulse responses of the same cells as in A normalized to maximum response. The vertical line shows the time at which the residual response (percent decrease from maximal response) was calculated as an index of the duration of response, as shown in Fig 3D.
C. Peak response amplitude of both ON and OFF cells as a function of the fluorescence intensity of the cell prior to visual stimulation.
D. Residual response of each cell at 4 seconds post stimulation, expressed as a fraction of peak response amplitude. This index shows the speed of response recovery towards the baseline, with brighter cells taking longer to recover.
(TIF)

S3 Fig Confocal SLO images of GCaMP expression in the primate tested in this study. A. 1 month after intravitreal injection of the viral vector. B. 21 months after intravitreal injection.
(TIF)

S4 Fig Illustration of high stability stimulus stabilization approach developed for this study. (TIF)
