# Supplementary material for: Functional architecture of the foveola revealed in the living primate
Source: PLoS One. 2018 Nov 28;13(11):e0207102. doi: 10.1371/journal.pone.0207102 (PMC6261564; doi:10.1371/journal.pone.0207102)
Supplement: S1 File — (DOCX) [file pone.0207102.s001.docx]

**Real time stabilization of the visual stimulus on retina**

As in earlier experiments^1^ the stability of fluorescent imaging of RGCs was achieved by using high fidelity reflectance imaging of cone photoreceptors to correct for eye movements when processing fluorescence data from the RGCs. However, this post processing procedure does not eliminate movement of the visual stimulus across photoreceptors. To stabilize the stimulus, the relative location of the stimulus with respect to individual cones is critical, thus we implemented an algorithm that predicted relative retinal location shift, and placed the visual stimulus accordingly. A related algorithm has been used in previous human and primate imaging studies^2–4^ for single cone stimulation, where the size of a stimulus is typically smaller than ~40μm or ~1/10 of the imaging field of view, and a single session of stabilized stimulation lasts less than 20 seconds. In this study the method was adapted to allow high stability functional imaging with a much larger stimulus, e.g., ~300μm x 300μm (256 x 256 pixels), spanning more than half the size of the imaging field of view, and for a much longer duration, e.g., ~200 seconds of continuous stabilization, and stabilization across multiple repeats

Due to potential in-frame image distortion, a large stimulus must be divided into multiple strips so that the motion of each strip can be calculated individually and the associated data is then played back through a field programming gate array (FPGA) to modulate the acousto-optic modulator (AOM) controlling the stimulation light source. This approach was described in detail for 10-15 second psychophysical experiments in the human eye^3^, however, it is likely to become inaccurate for continuous stimulation lasting 200-300 seconds. When a stimulus with 256 x 256 pixels is divided into 16 strips at 256 x 16 pixels/strip and the motion of each strip is calculated separately, the likelihood of algorithm error is increased more than 16 fold due to high variation of local image quality. To guarantee sufficiently accurate and robust stimulation, we developed an alternative, more conservative solution illustrated in Figure S4 below.

In Frame n of S4 Fig, stimulus to be played is shown as the light grey square, and the dark grey background is the imaging window. The fast scanner runs in vertical direction and the slow scanner runs in horizontal direction. The stimulus is split into two halves, the left strip ‘L’ and the right strip ‘R’. Data from area ‘a’ is used to calculate motion of strip ‘L’, and data from area ‘b’ is used to calculate strip ‘R’. When the size of ‘a’ falls below a user-defined critical value, e.g., 128 lines in the case here, the motion from the previous frame (Frame n-1) will be used to replace the motion of strip ‘L’. This large data set (‘a’ or ‘b’) substantially reduces uncertainly of local image quality and improves the robustness of motion calculation. This more robust solution does come at the prices of slightly decreased spatial accuracy of stimulation compared to the earlier solution^3^, but at 1 pixel r.m.s, the precision was adequate for the purposes of this study.

**References**

1. Yin, L. et al. Imaging Light Responses of Foveal Ganglion Cells in the Living Macaque Eye. J. Neurosci. **34,** 6596 (2014).

2. Sincich, L. C., Zhang, Y., Tiruveedhula, P., Horton, J. C. & Roorda, A. Resolving single cone inputs to visual receptive fields. Nat Neurosci **12,** 967–969 (2009).

3. Yang, Q., Arathorn, D. W., Tiruveedhula, P., Vogel, C. R. & Roorda, A. Design of an integrated hardware interface for AOSLO image capture and cone-targeted stimulus delivery. Opt. Express **18,** 17841–17858 (2010).

4. Arathorn, D. W. et al. Retinally stabilized cone-targeted stimulus delivery. Opt. Express **15,** 13731–13744 (2007).
