# Supplementary material for: Functional architecture of the foveola revealed in the living primate
Source: PLoS One. 2018 Nov 28;13(11):e0207102. doi: 10.1371/journal.pone.0207102 (PMC6261564; doi:10.1371/journal.pone.0207102)
Supplement: S2 File — (DOCX) [file pone.0207102.s002.docx]

**Long-term stability of GCaMP expression**

Typically G-CaMP expression levels in the primate fovea mediated by AAV with the ubiquitous CAG promoter show a reduction over a period of weeks to months, however in the case described here imaging was possible for more than a year. Figure 1B shows G-CaMP expressing cells near the conclusion of testing; a change in expression in the near foveal ring is frequently observed relative to the time of initial expression as shown in S3 Fig. Further work is required to understand how to balance titre, expression level, toxicity and immune response to achieve long term stability.
