# Supplementary material for: Functional architecture of the foveola revealed in the living primate
Source: PLoS One. 2018 Nov 28;13(11):e0207102. doi: 10.1371/journal.pone.0207102 (PMC6261564; doi:10.1371/journal.pone.0207102)
Supplement: S1 Table — (DOCX) [file pone.0207102.s003.docx]

**Table S1**: **Coefficients and 95% confidence intervals for least squares fits of 5^th^ order polynomial to RGC soma displacement as a function of receptive field eccentricity.**

$$f\left( x \right)=p_{0}+p_{1}x+p_{2}x^{2}+p_{3}x^{3}+p_{4}x^{4}+p_{5}x^{5}$$

|  | Nasal | Superior | Inferior | Temporal |
| --- | --- | --- | --- | --- |
| $p_{5}$ | 2.59e-7 ± 2.10e-7 | 1.13e-6 ± 1.34e-6 | -1.75e-6 ± 2.15e-5 | -1.51e-8 ± 4.95e-8 |
| $p_{4}$ | -6.99e-5 ± 5.02e-5 | -2.37e-4 ± 7.52e-4 | 2.62e-4 ± 3.21e-3 | 2.80e-6 ± 1.55e-5 |
| $p_{3}$ | 7.13e-3 ± 4.32e-3 | 1.89e-2 ± 2.10e-2 | -1.32e-2 ± 0.186 | 2.35e-4 ± 1.73e-3 |
| $p_{2}$ | -0.357 ± 0.163 | -0.747 ± 0.712 | 0.179 ± 0.182 | -7.38e-2 ± 8.34e-2 |
| $p_{1}$ | 10.8 ± 2.59 | 17.3 ± 10.7 | 5.95 ± 61.7 | 5.73 ± 1.68 |
| $p_{0}$ | 222 ± 13.3 | 140 ± 56.4 | 167 ± 292 | 216 ± 11.1 |
| $r^{2}$ | 0.924 | 0.861 | 0.870 | 0.911 |
| $f\left( 40 \right)$ | 386 ± 29.1 | 350 ± 27.8 | 337 ± 25.8 | 348 ± 24.4 |
