# Supplementary material for: Functional architecture of the foveola revealed in the living primate
Source: PLoS One. 2018 Nov 28;13(11):e0207102. doi: 10.1371/journal.pone.0207102 (PMC6261564; doi:10.1371/journal.pone.0207102)
Supplement: S2 Table — (DOCX) [file pone.0207102.s004.docx]

**Table S2. Numbers of ON- or OFF- center ganglion cells measured at different locations in fovea**

| **Location** | **Depth in GCL** | **Cell counts** | | | | |
| --- | --- | --- | --- | --- | --- | --- |
|  |  | ON-center | OFF-center | ON: OFF ratio | No impulse response | Total analyzed* |
| Nasal | Superficial | 120 | 24 | 5.0 | 71 | 215 |
|  | Deep | 61 | 32 | 1.9 | 70 | 163 |
| Temporal | Superficial | 46 | 43 | 1.1 | 90 | 179 |
|  | Deep | 82 | 76 | 1.1 | 42 | 200 |
| Superior | Deep | 71 | 44 | 1.6 | 102 | 217 |
| Inferior | Deep | 40 | 37 | 1.1 | 123 | 200 |

* Total analyzed: ON-, OFF- center ganglion cells and those that did not produced a statistical significant temporal impulse response for the RF center
