# Supplementary material for: Functional architecture of the foveola revealed in the living primate
Source: PLoS One. 2018 Nov 28;13(11):e0207102. doi: 10.1371/journal.pone.0207102 (PMC6261564; doi:10.1371/journal.pone.0207102)
Supplement: S3 Table — (DOCX) [file pone.0207102.s005.docx]

**Table S3. Configuration of imaging field and spatial resolution of visual stimulus**

| **Configuration of imaging field** | | **Spatial resolution of visual stimulus** | |
| --- | --- | --- | --- |
| Relative size | Width × height (degrees) | Check size in degrees | μm* |
| Large | 3.4 × 2.5 | 0.028 | 6.1 |
| Medium | 2.3 × 2.5 | 0.018 | 4.0 |
| Small | 1.4 × 1.5 | 0.012 | 2.6 |

* Conversion factor from visual angle to retinal distance is: 223 μm/degree
