# Supplementary figures and images for: Functional architecture of the foveola revealed in the living primate
Source: PLoS One. 2018 Nov 28;13(11):e0207102. doi: 10.1371/journal.pone.0207102 (PMC6261564; doi:10.1371/journal.pone.0207102)

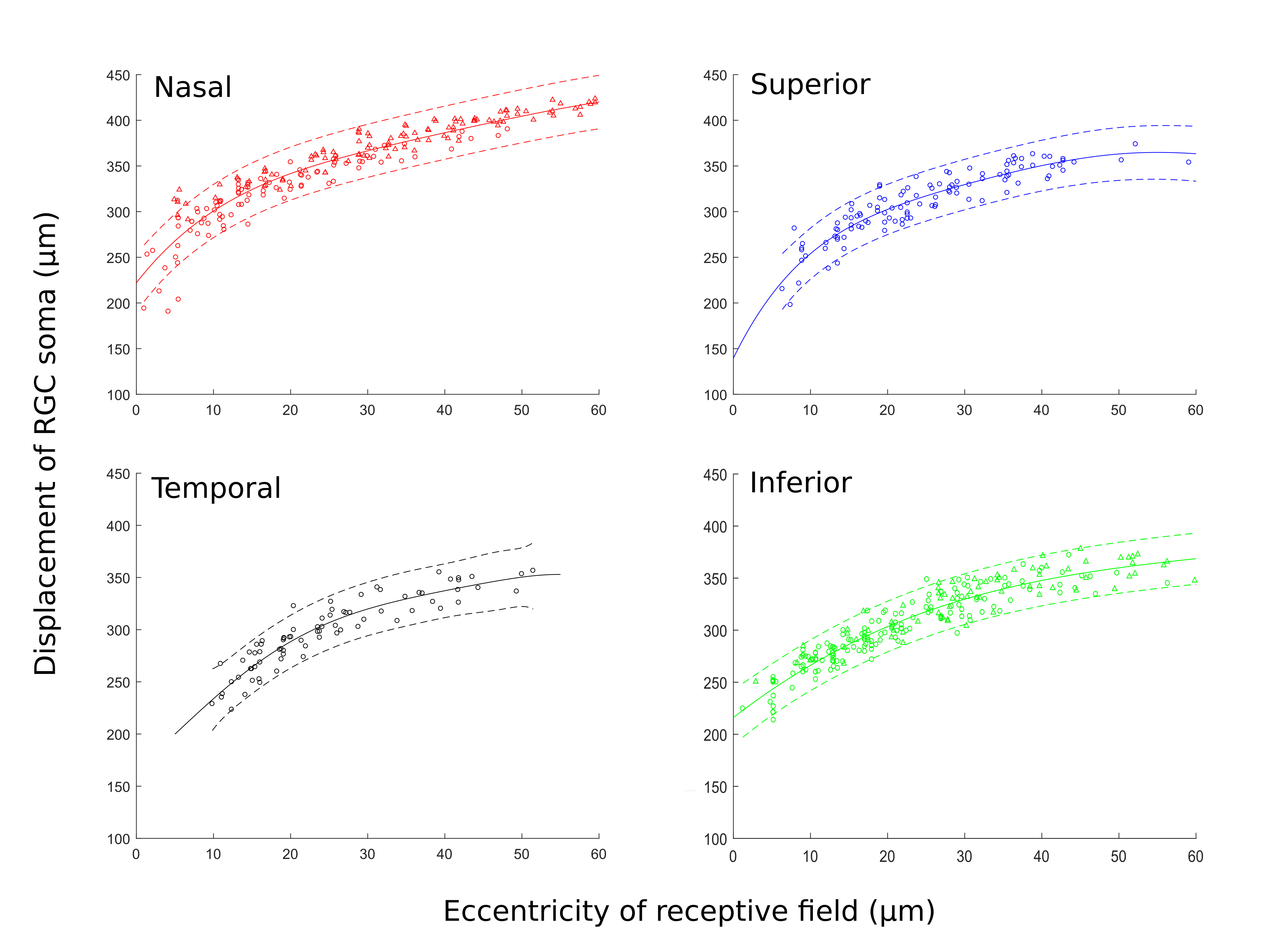

Supplement: S1 Fig — Triangles represent data taken at a superficial focus, circles represent data taken at the deeper focal position (see methods). Data were fitted with a 5th order polynomial, 95% confidence intervals are indicated by dashed lines. (TIF) [file pone.0207102.s006.tif]

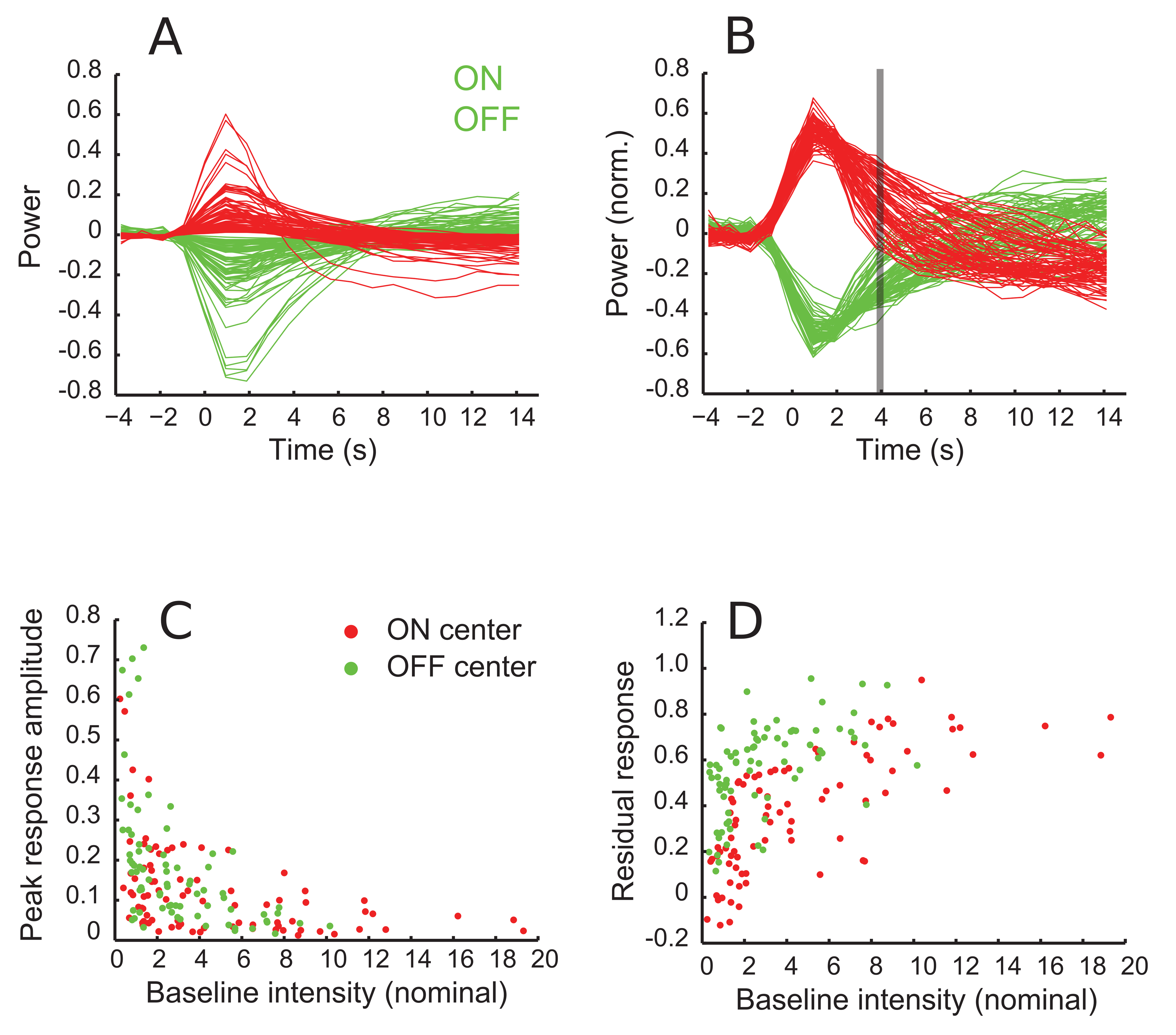

Supplement: S2 Fig — A. Impulse response of 40 foveal RGCs sorted into red ON responses (positive response to light stimulation) and green OFF responses (negative response to stimulation). B. Impulse responses of the same cells as in A normalized to maximum response. The vertical line shows the time at which the residual response (percent decrease from maximal response) was calculated as an index of the duration of response, as shown in Fig 3D. C. Peak response amplitude of both ON and OFF cells as a function of the fluorescence intensity of the cell prior to visual stimulation. D. Residual response of each cell at 4 seconds post stimulation, expressed as a fraction of peak response amplitude. This index shows the speed of response recovery towards the baseline, with brighter cells taking longer to recover. (TIF) [file pone.0207102.s007.tif]

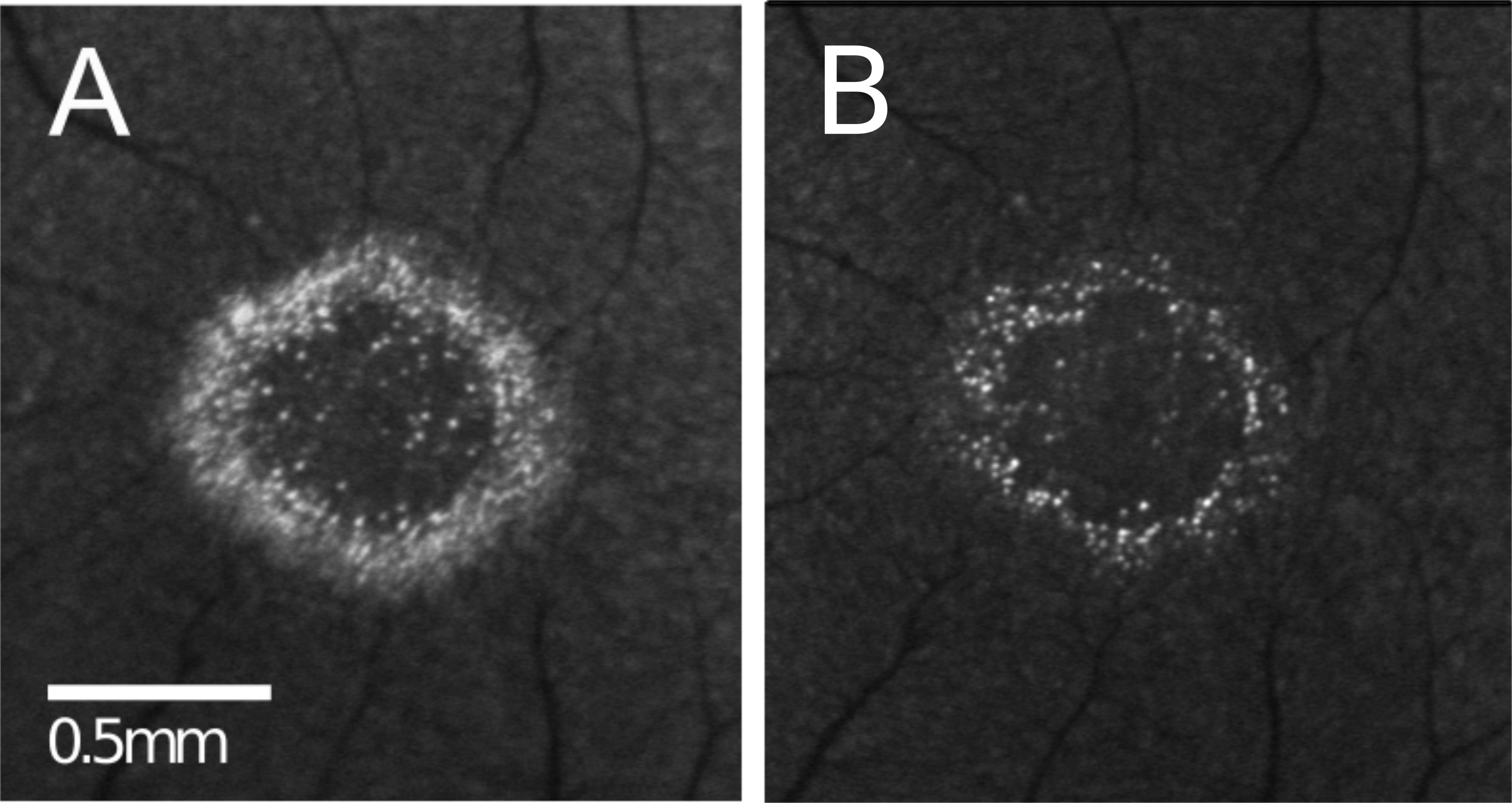

Supplement: S3 Fig — A. 1 month after intravitreal injection of the viral vector. B. 21 months after intravitreal injection. (TIF) [file pone.0207102.s008.tif]

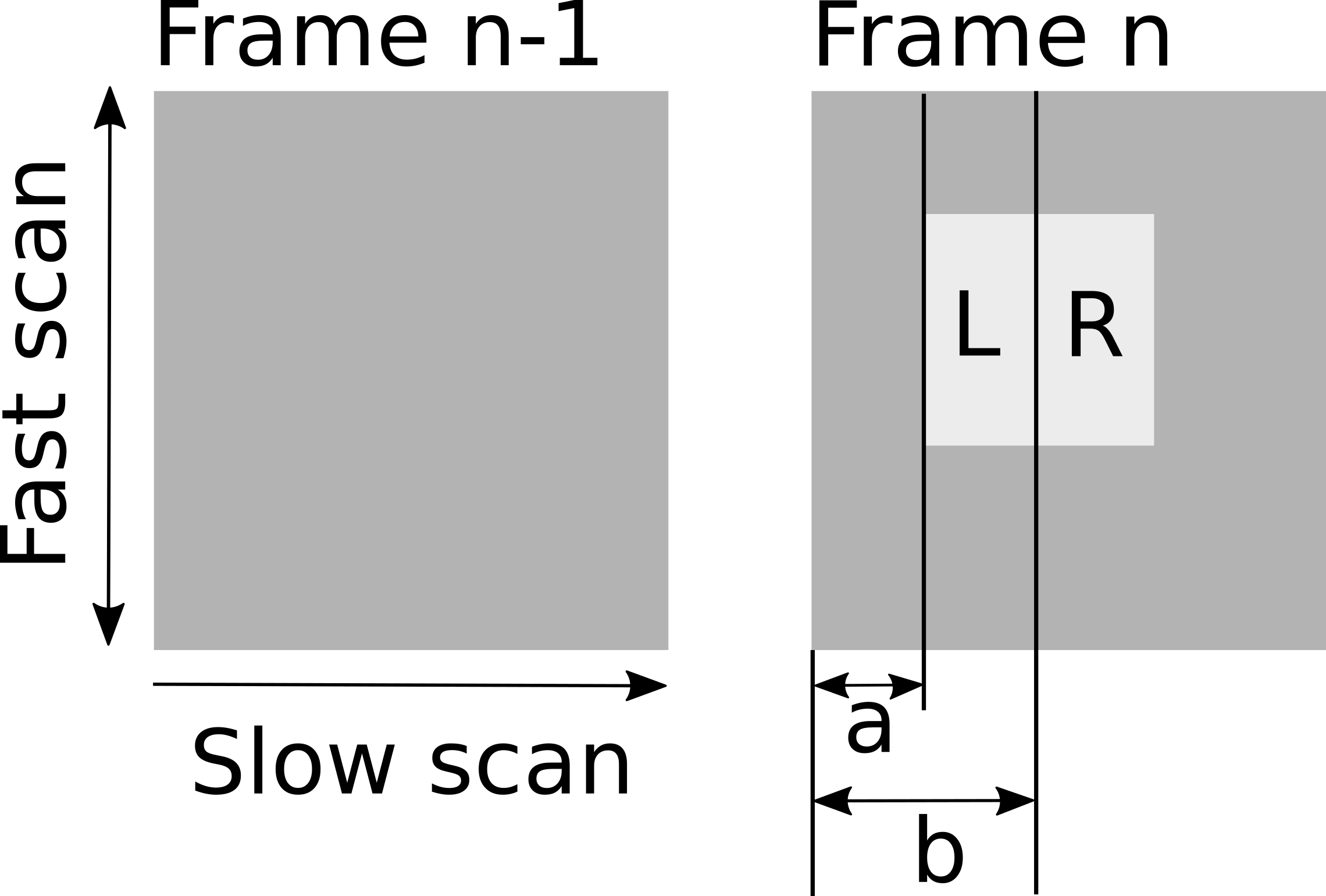

Supplement: S4 Fig — (TIF) [file pone.0207102.s009.tif]
